# Key quality indicators in colonoscopy

**DOI:** 10.1093/gastro/goad009

**Published:** 2023-03-10

**Authors:** Douglas K Rex

**Affiliations:** Division of Gastroenterology/Hepatology, Indiana University School of Medicine, Indianapolis, IN, USA

**Keywords:** colonoscopy, quality, adenoma detection rate, polypectomy, post-polypectomy surveillance

## Abstract

Many quality indicators have been proposed for colonoscopy, but most colonoscopists and endoscopy groups focus on measuring the adenoma detection rate and the cecal intubation rate. Use of proper screening and surveillance intervals is another accepted key indicator but it is seldom evaluated in clinical practice. Bowel preparation efficacy and polyp resection skills are areas that are emerging as potential key or priority indicators. This review summarizes and provides an update on key performance indicators for colonoscopy quality.

## Introduction

Colorectal cancer (CRC) is the second leading cause of cancer death in the USA, being the third leading cause in both men and women [1]. Colonoscopy plays an essential role in current efforts to reduce the incidence of and mortality from this common killer. In some countries, including the USA, colonoscopy is commonly used for primary screening [2]. In this context, colonoscopy is the only screening test that is recommended as infrequently as every 10 years [2–5] and evidence suggests that effective colonoscopy could be performed for screening as infrequently as every 15 [6] or 20 [7] years. In certain healthcare systems within the USA, and in many countries outside the USA, capacity and resources to offer and provide colonoscopy for screening are inadequate, and screening is performed primarily with fecal tests, usually the fecal immunochemical test (FIT) [8]. In the USA, both FIT and a combined FIT–multitarget DNA test are commonly used. Neither of these tests provides prevention of CRC unless positive tests are followed by colonoscopy, with identification of early cancers and effective identification and removal of precancerous lesions [9]. Throughout the world, colonoscopy is the test most commonly performed for patients presenting with colorectal symptoms and colonoscopy is the cornerstone of surveillance of patients with previous precancerous polyps or CRC [8].

Although multiple lines of evidence suggest that colonoscopy prevents CRC and CRC mortality, protection is imperfect and substantial evidence indicates that protection is highly dependent on colonoscopist performance [10–12]. Nearly every aspect of colonoscopy performance has been shown to vary, but the data are most robust for the detection of precancerous lesions [13–18]. The recognition of variable performance, as well as the impact of variable performance on important outcomes such as post-colonoscopy CRC (PCCRC), has led to a worldwide movement to improve the general performance of colonoscopy, make colonoscopy less operator-dependent, and thus improve patient outcomes [8]. Given the critical impact of colonoscopy on the prevention of colon cancer by identification and removal of precancerous lesions, it is clear that colonoscopy performed with high quality is crucial in every country with the resources to reduce CRC incidence, regardless of the approach that country takes to screening [8].

This review summarizes the history of the quality movement in colonoscopy, including recent evidence supporting changes to quality measurements for detection, as well as an update regarding the major or priority quality indicators. Measurement of the quality of resection has become increasingly the object of study, and of efforts to improve quality, and this topic is also covered.

## History of the quality movement in colonoscopy

Colonoscopes became commercially available and increasingly used in around 1970 and within a few years colonoscopy was widely taught within fellowship programs, and had been studied as a screening modality as early as 1990 [19, 20]. Despite this, awareness of variable performance did not begin to appear until the mid- to late 1990s [21, 22]. This largely reflects that detection during colonoscopy follows a rule that is generally true of human perception. That is, we are only aware of what we encounter with our senses, which in the case of colonoscopy means visualization of polyps. This allows anyone performing colonoscopy to believe they are extremely good at it, when in fact they might be an extremely poor performer.

In 1997, evidence appeared that the occurrence of PCCRC was significantly greater in some hospitals and with some colonoscopists than with others [21, 22] and the first large tandem colonoscopy study showed that colonoscopy missed 24% of all adenomas, 6% of large adenomas, and, most importantly for this discussion, the miss rate between individual endoscopists ranged from 17% to 48% [22]. This quickly led to widespread concern that some colonoscopy was performed carelessly, and perhaps too quickly. In 2002, the US Multi-Society Task Force on Colorectal Cancer proposed the first comprehensive set of quality indicators for colonoscopy, which included a new measure called the adenoma detection rate (ADR) [10]. The original recommendation was to measure colonoscopy in persons aged ≥50 years undergoing colonoscopy for reasons other than polyposis syndromes, inflammatory bowel disease, or positive fecal test. The ADR was proposed as a patient-based measure defined as the percentage of patients with at least one conventional adenoma detected and verified by pathology. In the same recommendations, the US Multi-Society Task Force recommended the minimum time for colonoscopy withdrawal in patients without biopsies or polyps should average 6–10 minutes [10]. The targets for ADR were initially set at ≥20% (25% in men and 15% in women) based on data from available screening colonoscopy studies [23–26]. These targets were not set at the maximum rates of detection, but at just below the mean prevalence observed in available combined studies. Thus, the initial proposal was based on the unproven consideration that raising the performance of the worst performers would have the greatest impact, and not setting the acceptable threshold so high that nearly all colonoscopists would be inadequate. The recommended withdrawal time was based on minimal evidence. In the above-mentioned tandem study [22], the two colonoscopists with the lowest observed miss rates had average withdrawal times in normal colons of ∼8 minutes. This led to the original 6- to 10-minute recommendation.

In 2006, the task of creating quality indicators for the USA fell to a new combined task force of the American College of Gastroenterology (ACG) and American Society for Gastrointestinal Endoscopy (ASGE) [11]. This year coincided with a landmark study published from a group of private practitioners in Rockford, IL, USA, showing a tight correlation between ADR and withdrawal time, and indicating that 6 minutes of withdrawal time in normal colons provided reasonable separation between high and low ADR performers [13]. In response to this, the ACG–ASGE changed the recommended minimum withdrawal time to 6 minutes [11]. Because the original target of 20% had been set based on screening colonoscopy studies, the ACG–ASGE Task Force also recommended that ADR measurement be confined to first-time screening colonoscopies [11].

The first validation of ADR as a predictor of interval cancer was published in 2010. In a Polish screening colonoscopy study, the patients of doctors with ADRs of <20% had hazard ratios ≥10 times higher for PCCRC compared with patients of doctors with ADRs of >20% [15]. There were too few cancers appearing in patients of doctors with ADRs of >20% to determine whether increasing the ADR to >20% was of value.

In 2014, data from a study with many more PCCRCs showed that PCCRC reductions did progressively increase with ADRs of >30% [16] and, in 2015, the ACG–ASGE Task Force responded by raising the minimum threshold for ADR to 25% [12]. In addition, ADR was recommended as the preferred indicator of quality, since prospective studies with withdrawal time did not consistently show that increasing withdrawal time improved detection [27]. Short withdrawal time was suggested to be used as an indicator of poor technique in colonoscopists with ADRs below the minimum threshold.

Outside the USA, important contributions were made in Europe, including in the UK [28], Poland [29], and Germany [30]. In particular, these studies emphasized aspects of colonoscopist training that improved ADR [29] and the colonoscopy experience for patients [28].

Within the USA, the development of large registries for practicing physicians to benchmark their performance became warehouses of large amounts of important data, particularly regarding detection [31].

A new development in the 2015 ACG–ASGE Task Force recommendations was the presentation of priority quality indicators that should be measured by everyone performing colonoscopy. The initial recommended priority indicators were ADR, cecal intubation rate (CIR), and appropriate use of screening and surveillance intervals [12].

Since 2015, new evidence has suggested the potential to make further refinements in ADR. In response to lowering the recommended screening age from 50 to 45 years in the USA, several studies showed that the prevalence of adenomas in 45- to 49-year-olds is only slightly below that in 50- to 54-year-olds, suggesting that ADR measurements could be lowered to include 45- to 49-year-olds without adjustments in the recommended threshold [31–39].

Second, several studies evaluated the incidence of adenomas 10 years after a negative screening colonoscopy and showed that it was only slightly below the prevalence of first-time screening colonoscopies [40–44]. These data indicate that subsequent screening colonoscopies after the initial could be included in the ADR measurement.

Third, data from large registries showed that ADR progressively increased in the USA after 2010 and by 2018 had risen to 39% [31]. Further, a large study from northern California Kaiser showed that increasing ADR continued to provide additional protection against PCCRC, even as the ADR increased to >40% [45]. The Kaiser group, whose 2014 study had shown that for each 1% increase in ADR, there was a 3% reduction in PCCRC incidence, and a 5% reduction in PCCRC mortality [16], showed that the same rule applied during a later interval when ADRs were generally higher [45]. These data support an additional increase in the minimum acceptable ADR threshold.

Fourth, these data showing continued improvement in PCCRC rates as ADR rises suggest that while a minimum acceptable threshold is appropriate, even doctors with ADRs above this minimum threshold should strive to improve detection. This suggests the concept of an aspirational threshold [46], which might approach 50% for primary screening [47].

Fifth, several studies found that inclusion of patients undergoing surveillance (those with previous adenomas or cancer) or diagnostic (colonoscopy performed for symptoms) could be included in the ADR measurement without substantially affecting the risk of individual physicians falling above or below the minimum recommended threshold [44, 48–57]. Surveillance colonoscopy is associated with an ADR of 7%–12% above screening, though screening is typically higher than diagnostic, so that the average of all three groups of indications generally approximates the screening result. Inclusion of large numbers of patients makes measurement of ADR easier and results in an ADR with a narrower confidence interval around the ADR measurement. If surveillance and diagnostic examinations are included, there would still be the need to exclude patients with positive fecal tests, whether by FIT or FIT–multitarget DNA, and it would still be necessary to exclude inflammatory bowel disease and polyposis syndromes.

These new types of evidence indicate that ADR definitions and recommendations around measurement are likely to continue to evolve.

Thus far, recommendations for quality measurement have had little to say with regard to resection—an important part of colonoscopy that, when ineffective, can contribute to PCCRC [58]. As evidence regarding the measurement of quality of resection improves, this is likely to be the next major focus of quality recommendations.

## Adenoma detection measures for colonoscopy

Table 1 lists a series of detection measures for conventional adenomas based on their likely relationship with the central goal of most colonoscopies, which is the prevention of CRC. In creating a usable detection measure, important considerations have included clinical relevance, feasibility of measurement, and resistance of the measure to corruption or gaming [59].

**Table 1.** Potential indicators of adenoma detection listed from the most directly related to cancer prevention to the least directly related

Indicators
Post-colonoscopy colorectal cancer rate (PCCRC rate)
Advanced adenoma miss rate (AAMR)
Adenoma miss rate (AMR)
Advanced adenoma detection rate (AADR)
Adenomas per colonoscopy (APC)
Adenoma detection rate (ADR)
Polyp detection rate (PDR)

As seen in Table 1, it is clear that ADR is not the measure that is logically most closely associated with prevention of PCCRC. However, ADR has reasonable feasibility of measurement, though imperfect. This imperfection in feasibility is related to the need to populate quality databases with pathology information to confirm that resected polyps are conventional adenomas. Although some programs linking pathology databases and endoscopy data directly have been used, and other programs have involved natural language processing of endoscopy and pathology reports [60], in clinical practice, manual entry of pathology databases has most often been required. A strength of ADR is its resistance to gaming, based on ADR being a total colon (rather than restricted to some portion of the colon) measurement. Most importantly, there is clear evidence that pathologists are consistently effective at categorizing polyps into the conventional adenoma vs serrated class. The only common exception is the traditional serrated adenoma, which is often interpreted in clinical practice as a tubulovillous adenoma, but is also a rare lesion with little impact on detection measures [61]. One form of potential gaming of ADR is “one and done” behavior. Thus, since ADR is a patient-based measure, it is possible for an endoscopist to remove one adenoma and not check the rest of the colon carefully. Despite the enormous available literature on ADR, only one clear instance of one and done has been observed [62], and it likely involved gaming of the US reimbursement system rather than ADR, since US endoscopists are generally paid only for the first polyp removed, and the involved endoscopists were not aware that ADR was being measured. Another potential risk of ADR is “indication gaming” in which a patient presents with symptoms and is eligible for screening, and the physician decides after the procedure whether to use the symptom or screening as the primary indication, with awareness of whether or not an adenoma was detected [48]. Elimination of the potential for indication gaming would be an advantage of returning the ADR to include surveillance and diagnostic examinations. A final advantage of continuing to use ADR is that the measure is now well validated as a predictor of PCCRC.

Adenomas per colonoscopy (APC) or one of its variants deserves careful consideration as a substitute for ADR. APC has the advantage of considering every adenoma detected, and therefore incentivizes full and complete clearing during colonoscopy. Despite these advantages, the extent to which APC would improve prediction of PCCRC compared with ADR is not clear. There are practical disadvantages to APC, including potentially incentivizing placing individual polyps in separate bottles for pathology, which would increase costs. An alternative policy of photography of each lesion had been described [63], but this practice is not in widespread use and is still beyond the capability of some endoscopy units. APC might also be more difficult, time-consuming, and resource-intensive to calculate compared with ADR, since it would require confirmation of each lesion removed as an adenoma.

The most direct and relevant measure is the rate of interval PCCRCs. Use of this as the primary measure of detection has been resisted since the confidence interval around the measurement would be wide for colonoscopists with low procedure volume and it might take several years or more of observation to recognize an underperforming endoscopist.

The adenoma miss rate (AMR) and advanced adenoma miss rate (AAMR) have the clear disadvantage of requiring tandem colonoscopies to measure, which is impractical in clinical practice [64]. The advanced adenoma detection rate (AADR) often correlates well with the ADR and is sometimes suggested to be more relevant to PCCRC prevention. However, prospective measurement of advanced adenomas, and to a significant extent retrospective measurement, is subject to significant biases, including variable polyp size estimation by endoscopists and wide interobserver variation among pathologists in the diagnosis of villous elements and dysplasia grade [65].

The polyp detection rate is a very practical measure that correlates well with ADR in retrospective studies [66]. However, its effective prospective use in quality programs is not established and logically could incentivize the removal of the ubiquitous distal colon diminutive hyperplastic polyp, which is still widely considered to not be precancerous.

Given these considerations, ADR remains the likely choice to continue as the primary measure of conventional adenoma detection, with APC an alternative that could receive widespread implementation or, alternatively, implementation within individual institutions. Most data suggest that an APC of ∼0.6 correlates with current ADR minimum thresholds [60]. An APC of <0.6 would warrant remedial corrective work and APCs rise to well over 1 in high-level detectors [67].

Higher ADRs are needed in colonoscopy programs that focus on FIT testing. In general, the ADR cut-offs for an acceptable threshold should be 15%–20% higher than for ADRs expected in primary screening colonoscopy [9]. However, expected ADRs in a FIT-based colonoscopy program will increase with higher thresholds of hemoglobin content in the feces required for a positive FIT test [9]. Variation in fecal hemoglobin levels used across the world makes it difficult to determine an internationally appropriate cut-off for minimum acceptable ADR in a FIT-positive colonoscopy screening population. It is clear that while surveillance and diagnostic examinations for symptoms might be included in a new definition of ADR, inclusion of FIT-positive or FIT-DNA-positive patients will skew the calculation in an unacceptable fashion and these patients should be excluded from primary screening ADRs, along with patients with inflammatory bowel disease and polyposis syndromes.

## Serrated indicators

Current recommendations are that serrated lesions be excluded from the ADR calculation [12]. The rationale has been the difficulty in establishing a workable serrated target. Differentiation of a sessile serrated lesion from a hyperplastic polyp has a long history of high interobserver variation between pathologists [68], with evidence suggesting that the pathologists in some centers never diagnose sessile serrated lesions [69]. To overcome this, a combined target of sessile serrated lesions and proximal hyperplastic polyps can be created, but it is not clear that this would work in prospective use, as it might incentivize physicians to divide the colon into proximal vs distal location at a more distal location than they otherwise would, in order to include distal hyperplastic polyps.

Despite these challenges of establishing a serrated target, evidence has arisen that a small fraction of colonoscopists have high ADRs and are low serrated detectors [70, 71], and that low serrated detection is an independent predictor of PCCRC [70, 71], thus the rationale for creating an independent serrated target, or merging a serrated detection target with conventional adenoma detection.

Table 2 lists various potential serrated detection indicators. The simplest for implementation would be a sessile serrated lesion detection rate (SSLDR). The SSLDR would have the disadvantage already mentioned of interobserver variation between pathologists, but it could force endoscopists to involve their pathologists in education efforts and cooperation to embrace identification of sessile serrated lesions (SSLs).

**Table 2.** Potential indicators of serrated lesion detection quality

Indicators
Sessile serrated lesion detection rate (SSLDR)
Clinically significant serrated polyp detection rate (CSSPDR)
Proximal serrated polyp detection rate (PSPDR)
Total serrated polyp detection rate (SPDR)

A total colon serrated polyp (hyperplastic polyps plus SSLs) usually raises the objection of incentivizing removal of distal colon diminutive hyperplastic polyps. The proximal serrated polyp detection rate (PSPDR) has been shown to be a predictor of PCCRC [71] and in prospective use would be subject only to potential gaming of polyp location within the colon.

Most of the other potential indicators (Table 2) are subject to both polyp size measurement bias and location bias, and would be unlikely to hold up in prospective use.

## Improving ADR

Recent evidence indicates that ADR can be improved, with a result that the patients of physicians with low ADRs experience better protection against PCCRC after physician improvement [29]. The fundamentals of detection are the ability to recognize lesions when they appear on the television monitor, and effective exposure of all colonic mucosa, so that the opportunity for recognition by the endoscopist occurs (Table 3).

**Table 3.** Measures that increase adenoma detection

Level	Measures
Pre-procedure	Fully trained and committed colonoscopist
	Split or same-day preparation
	Measurement and reporting
Technique-based	Intraprocedural cleansing
	Adequate distension
	Detailed mucosal exposure
	Double examination of the right colon
	Water exchange
	Patient rotation to keep examined segment non-dependent
Highlighting tools	High-definition colonoscopes
	Artificial intelligence
	Pan-colonic dye spraying (chromoendoscopy)
	Methylene Blue (MMX)
	Electronic chromoendoscopy
	Narrow-band imaging
	Linked color imaging
	Blue light imaging
	i-scan
Mucosal exposure tools	G-Eye balloon
	Distal attachment, cap, or hood
	Computer-assisted quality (CAQ) program
	Third-eye retroscope

Important pre-procedure steps include split or same-day bowel preparation [16], which has improved lesion detection in randomized–controlled trials [72], use of high-definition colonoscopes [73], and institution of a systematic process for ADR measurement and reporting [74]. Thus, feedback alone produces gains in ADR. A video-recording study, in which colonoscopies were video recorded without the knowledge of endoscopists, followed by video recording with the endoscopists’ knowledge, showed convincingly that endoscopists generally understand the principles of effective examination, but may not exercise these principles without monitoring [75]. The ultimate expression of monitoring, continuous video recording of all examinations, is now available but still not in widespread use.

During the colonoscopy procedure, the core of effective detection is readily expressed as compulsive examination of the proximal sides of folds, flexures, and valves; cleaning of mucosal surfaces; and achieving adequate distension [76]. In one study, intraprocedural cleaning accounted for 17% of the procedure time and raised the rate of adequate bowel preparation from 90% before intraprocedural cleaning to 96% after intraprocedural cleaning [77]. Intraprocedural cleaning occasionally exposes polyps hidden under pools of debris and also increases the hover time of the colonoscope tip. In the left lateral decubitus position, which is typically the position for withdrawal, distension can become problematic in the left colon, which is dependent. Distension in the left colon can be achieved by rotating the patient out of the left lateral decubitus position [78], filling the left colon with water rather than air, use of a balloon device that occludes the colon distal to the colonoscope tip [79], and manual pressure on the patient’s buttocks to prevent gas escape from the anus. Gas escape seems particularly problematic in patients sedated with propofol and/or those with an incompetent anal sphincter. The use of carbon dioxide creates more willingness on the physician’s part to aggressively insufflate, since there is little concern about pain in the recovery area from abdominal distension.

Several specific techniques have been associated with improved detection. These include patient rotation [80], which improves detection through more effective distension [78]. This technique generally requires a patient who is not in deep sedation or general anesthesia, in order to avoid the risk of aspiration associated with rotation into the supine or right lateral decubitus position, as well as injury to endoscopy workers from rotating obese patients who are too sedated to assist. A second technique associated with improved ADR is water exchange [81], in which during the insertion process water is used to fill the colon and exchanged for dirty water, with the colonoscope tip moving forward only after full exchange. In clinical practice, this process is best performed with saline, since use of actual water induces the production of thick mucus during withdrawal that itself obscures visualization [82]. Water exchange studies have been largely concentrated from a few centers and many experts consider the process too inefficient for routine use. Water exchange, as well as simple water filling during insertion, does increase patient comfort when the procedure is performed unsedated or with minimal sedation [83, 84]. A third important technique is double right colon examination. Colonoscopy has been consistently less effective in preventing right-sided colon cancers compared with left-sided colon cancers [85–89]. Thus, many experts advocate an initial examination of the right colon in the forward view from the appendiceal orifice to the hepatic flexure, followed by reinsertion of the colonoscope and re-examination of the cecum and right colon in the forward view, or the cecum in the forward view plus examination of the right colon in retroflexion [90–93]. Randomized–controlled trials show no significant difference between the second examination performed in the forward view vs retroflexed, though numerically the results favor a second forward examination [91]. However, the key is to perform two examinations of the right colon in most or all patients.

Adjuncts to detection can be divided into those that enhance mucosal exposure and those that highlight flat and subtle lesions for endoscopist recognition. Among the mucosal exposure devices, the Endocuff (Olympus Corporation, Center Valley, PA) is the best studied and validated, with an overall improvement in ADR of ∼7% [94, 95]. In one randomized–controlled trial, the Endocuff Vision (ECV), the more recent model of the device, allowed withdrawal ∼2 minutes faster than standard colonoscopy, but still with numerically improved detection [96]. A cap or hood on the colonoscope tip is an alternative to Endocuff and is generally easier to pass through a narrowed sigmoid colon, and allows easier intubation of the terminal ileum [97]. Finally, AI programs that evaluate the quality of inspection and provide immediate feedback have resulted in substantial gains in detection [98, 99] and gains that are additive to AI-based highlighting programs (CADe).

A variety of tools can enhance the detection of subtle lesions. These include pancolonic dye spraying or chromoendoscopy [100, 101], electronic forms of chromoendoscopy including narrow-band imaging [102] (NBI; Olympus Corporation, Center Valley, PA), blue light imaging (Fujifilm, Valhalla, NY) [103], and linked color imaging (Fujifilm, Valhalla, NY) [104]. NBI did not appear effective for detection using the Olympus 180-series colonoscope, but was effective in studies utilizing the 190-series colonoscope, which has brighter colonic illumination in the NBI mode [102].

The largest gains in detection have occurred with artificial intelligence highlighting programs (CADe). Now tested in multiple parallel arm studies and tandem studies [105–107], CADe programs have produced gains in ADR of ∼10%, gains in both low and high detectors, and reductions in missing of ≥50% in tandem studies [105–107]. There is evidence that CADe programs are complimentary both with computer-assisted quality (CAQ) AI programs [98] and with the use of ECV.

In summary, the modern high-level detector approaches colonoscopy with a thorough understanding of the range of appearances of precancerous lesions in the colon, a high-definition colonoscope, split or same-day bowel preparation, an established ADR measurement and reporting system, and an applied detailed examination technique, ideally using both a mucosal exposure device and a CADe program to enhance detection.

Table 3 does not include the measurement of withdrawal time as an effective way to improve detection. Although withdrawal time correlates well in retrospective studies with lesion detection and even with PCCRC prevention, the prospective use of withdrawal time to improve detection is inconsistently effective [27]. Corrective measures should focus on the application of effective technique, which when performed will result in longer withdrawal time. Recent evidence suggests that a withdrawal time of ∼9 minutes optimizes the detection of both adenomas [108–122] and serrated lesions [120, 121, 123] and prevention of cancer [18]. In clinical practice, withdrawal time should be measured, and a low ADR or inadequate serrated detection should be used as a signal of inadequate technique and/or ineffective lesion recognition. A short withdrawal time in a low detector suggests the need for evaluation of multiple parameters to potentially improve performance, but most importantly, the need for application of an effective withdrawal technique.

## CIR

Cecal intubation is defined as advancement of the colonoscope tip proximal to the ileocecal valve so that the appendiceal orifice and entire medial wall of the cecum is evident for inspection. Recent recommendations have been that cecal intubation should be achieved in ≥90% of overall colonoscopies and ≥95% of screening colonoscopies [12]. In US gastroenterology practice, overall cecal intubation rates are typically well above 95%. Low cecal intubation rates have been associated with increased risk of PCCRC [124]. Recommendations have been to accompany cecal intubation by adequate photo documentation of the ileocecal valve, and terminal ileum if intubated [12]. Cecal landmarks identified and those that are photographed should also be noted in the colonoscopy report.

The CIR has been considered a priority quality indicator, but very high levels of performance that tend to remain stable or increase over time, but not decrease, have raised the question of whether the targets are too easy to achieve. Perhaps this target is too consistently achieved to maintain clinical relevance over time. Intermittent checks of cecal intubation rates could be sufficient to document adequate performance, especially in those with high colonoscopy volumes [125].

## Bowel preparation indicators

A number of studies suggest that individual centers have continued to have rates of inadequate bowel preparation during colonoscopy of 20%–25% [126, 127]. These rates are costly, with the need for repeat procedures increasing the total cost of delivering colonoscopy by ∼1% for each 1% of examinations for which the preparation is inadequate [128].

These high costs of repeat procedures and patients lost to follow-up suggest that bowel preparation quality could be a priority indicator for colonoscopy. In 2015, the US Multi-Society Task Force recommended that ≥85% of examinations in outpatients should have an adequate preparation [129] and the European Society for Gastrointestinal Endoscopy recommended that adequate preparation should be achieved in ≥90% of colonoscopies [130].

Bowel preparation quality should be judged after intraprocedural cleaning. The best validated cleansing score for clinical use is the Boston Bowel Preparation Score [131–133] and a score of ≥2 in each colon segment correlates with adequate preparation. Patients with these scores should have intervals for the next screening or surveillance examination that are consistent with current screening or post-polypectomy surveillance guidelines. Patients with inadequate preparation should have their procedure repeated within 1 year [129].

A variety of clinical practices are useful in improving patient satisfaction associated with bowel preparation. For example, patients with brown effluent on the day of presentation should be considered for additional preparation at the endoscopy suite before colonoscopy is initiated, since a brown effluent indicates a 50% risk of inadequate preparation [134]. Patients with predictors of inadequate preparation including previous colon resection, chronic constipation, use of constipating drugs such as opioids or tricyclics, obesity, or diabetes should be considered for additional preparation doses [135]. Patients who are non-native English-language speakers, or have poor socioeconomic status, are more successful when the bowel preparation process is navigated [135].

## Resection

Although the main cause of PCCRC is missed lesions [136], there is some evidence that ineffective lesion resection contributes to PCCRC [58, 136, 137]. In addition, assessments of complete resection performed by biopsy of the margins and/or center after resection have indicated that rates of incomplete polyp resection vary by 3-fold between endoscopists [138]. Recognition of this variation has led to recommendations to assess and measure the quality of technical performance of resection.

Two validated scales for measuring the technical quality of resection have appeared. The first and more thorough of these is the Direct Observation of Polypectomy Skills (DOPyS) [139]. The DOPyS scale assesses resection quality of a range of lesions from small diminutive to larger lesions requiring endoscopic mucosal resection (EMR). Early assessments suggested that competency in technical resection varied 3-fold, with some endoscopists performing technically competent resections in only 30% of cases [140], and that there was no good correlation between detection skill as measured by ADR and competency in resection [140]. Common errors included failure to fully assess the lesion pre-resection, failure to position the lesion in the 5 to 6 o’clock portion of the endoscopic field, failure to maintain an appropriate working distance, failure to place the snare adequately, and failure to assess the resection margin after removal [140].

A shortened version developed specifically for assessment of cold snaring, which can now be used to effectively remove ≤90% of lesions, is the Cold Snare Assessment Polypectomy Tool (CSPAT) [141]. This tool may be more appropriate for many clinicians in practice who do not perform complex resections, and for assessing and training gastroenterology fellows.

Both DOPyS and CSPAT can be applied by real-time in-room monitoring by an assessor, or by assessment of video recordings. Both methods are labor- and resource-intensive for use in clinical practice, though each merits strong consideration by institutions, and each can serve as the basis of effective polyp resection teaching during gastroenterology fellowship.

Other simpler approaches to important outcomes can be assessed by audit. For example, current US [142] and European [143] guidelines recommend that benign lesions of any size generally not be referred for surgical resection. Surgical resection of benign lesions is associated with greater mortality, morbidity, and cost compared with endoscopic resection. All benign lesions should be reviewed by an expert colorectal endoscopic resectionist prior to referral to surgery in order to reduce overuse of surgical resection for benign lesions. Recent audits in the USA suggest that surgery for benign lesions has only recently started to decline [144].

Another potential candidate for audit review is the use of cold forceps to remove lesions of >3 mm in size. The US Multi-Society Task Force had recommended that cold forceps not be used for lesions of >2 mm [142] and the European Society for Gastrointestinal Endoscopy recommends that forceps not be used for lesions of >3 mm [143]. Cold forceps resection of lesions of >3 mm is associated with incomplete resection and is less efficient than snare resection. Despite that, there is evidence in clinical practice that use of cold forceps to resect lesions of >3 mm remains widespread [145]. Inappropriate use of cold forceps resection is easily subject to audit review.

## Screening and surveillance intervals

The use of appropriate screening and surveillance intervals was established as a priority quality indicator for colonoscopy in 2015, with 90% set as the adherence target [12]. Overuse of colonoscopy in low-risk patients and overuse in high-risk patients have been previously summarized [12]. Surveillance practice in the USA is dominated by the US Multi-Society Task Force [146] recommendations. Overuse of colonoscopy for surveillance [147] and screening [148] since the 2015 quality recommendations is well documented. Appropriate use of screening and surveillance is important to optimizing the cost-effectiveness of colonoscopy.

## Other indicators

US quality recommendations cover a broad range of other topics related to the technical performance of colonoscopy and review of these recommendations is important to the quality of colonoscopy [12]. Many of the recommended indicators are seldom measured in clinical practice. An important consideration is whether the long-term measurement of priority indicators like ADR and CIR is appropriate for physicians who have consistently achieved high-level performance and are unlikely to have declines in performance [125, 149]. Thus, stopping the measurement of time- and resource-intensive end points for colonoscopists who have consistently demonstrated high performance, followed by use of those resources to explore adherence to other quality indicator targets, is a rational and evidence-based strategy.

## Conclusions

Effective colonoscopy consists of high-quality bowel preparation, safe and complete colonoscope insertion to the cecum, high levels of detection, and effective resection of all detected benign lesions without the use of surgery. Variable performance with regard to these parameters is common with continued enormous room for improvement in clinical practice. Colonoscopists of all specialties should measure the priority quality indicators, and institute remediation and improvement whenever deficiencies are identified. Public transparency of quality indicators remains an important goal.
